# ‘Comment on: Livestock farmers’ knowledge, attitudes and practices relating to zoonoses in the Coastal Savannah zone of Ghana’: reply

**DOI:** 10.1093/inthealth/ihaf135

**Published:** 2025-11-18

**Authors:** Sylvia Afriyie Squire, Godwin Yao Ameleke

**Affiliations:** Animal Health Division, Council for Scientific and Industrial Research – Animal Research Institute, P.O. Box AH 20, Achimota, Accra, Ghana; Department of Natural Resources Management, CSIR College of Science and Technology, P.O. Box AH 20, Achimota, Accra, Ghana; Department of Natural Resources Management, CSIR College of Science and Technology, P.O. Box AH 20, Achimota, Accra, Ghana; Livelihood and Innovation Division, Council for Scientific and Industrial Research – Animal Research Institute, P. O. Box AH 20, Achimota, Accra, Ghana

Dear Editor,

We appreciate the opportunity to respond to comments on our paper, ‘Livestock farmers’ knowledge, attitudes and practices relating to zoonoses in the Coastal Savannah zone of Ghana', published in *International Health*.^1^ We appreciate Davey’s interest in our work and for highlighting the need for immediate action based on our findings and recommendations.^2^ We welcome the scrutiny, critique and suggestions for future studies,^2^ which enrich the discussion on this critical topic. In this response, we address key concerns and provide clarification on several points.

We acknowledge Davey’s observation that the 2023 anthrax outbreak in Ghana should have led to improved understanding among respondents.^2^ However, as indicated in our study design, our survey was conducted in 2022,^1^ prior to this outbreak. Consequently, it was unlikely that respondents would have acquired knowledge from this specific event. The observed low knowledge about anthrax in the coastal savannah zone aligns with the disease’s endemicity in northern Ghana, outside our study area.^1^ Further research is needed to investigate how the 2023 outbreak and interventions influenced awareness related to anthrax among Ghanaians, particularly livestock farmers and rural communities nationwide, given the outbreak’s effective management using a One Health approach and rapid containment.^3,4^ We support recommendations for education campaigns on anthrax and other livestock-related zoonoses where necessary, and further interventions including responsible animal farming practices, improved surveillance and early diseases detection, diagnosis and treatment,^2,5^ emphasizing the importance of a tailored approach, as highlighted in our study.^1^

Regarding the study design, we acknowledge the suggestion that differentiating between knowledge, perceptions, attitudes and practices could have been more explicit. Due to the word limit for research papers in *International Health*, we condensed our descriptions of conceptual and operational definitions and measurements. Nevertheless, our results provide comprehensive information on the questions posed to farmers, and the data analysis section outlines the transformations carried out to measure our constructs of knowledge, attitude and practice.^1^

We agree that analyzing the relationships between attitude and practice, and knowledge and practice, beyond descriptive statistics, would yield valuable insights. However, this analysis was beyond the scope of our current paper, which focused on examining the relationship between farmers' socioeconomic and farm characteristics and their knowledge, attitudes and practice regarding zoonosis using logistic regressions. Understanding how these attributes influence knowledge, attitude and practice would provide a foundation for designing effective interventions to promote good practices and reduce zoonoses. We plan to consider this analysis in future studies.

Additionally, we concur with the importance of identifying potential targets for evidence-based interventions aimed at changing attitudes and behaviors. In line with this, our paper recommends targeting farmers in extensive production systems for zoonoses-prevention initiatives, including targeted education on lesser known zoonoses such as anthrax and brucellosis, as well as training on reverse zoonoses transmission and proper disease-management practices.^1^

Finally, we appreciate Davey’s suggestions for future studies, including combining quantitative and qualitative data to provide in-depth information on human–livestock interactions, slaughtering and butchering of infected animals, availability of veterinary services and gender inequality, as well as exploring the implications of culture, traditions and indigenous health beliefs for understanding or curbing zoonotic transmission.^2^ We will seek opportunities to pursue these research directions with the necessary resources.

## Data Availability

No new data was generated for this correspondence.
